# Quality of Life and Medication Adherence of Independently Living Older Adults Enrolled in a Pharmacist-Based Medication Management Program

**DOI:** 10.3390/pharmacy5020020

**Published:** 2017-04-06

**Authors:** Christopher Harlow, Catherine Hanna, Lynne Eckmann, Yevgeniya Gokun, Faika Zanjani, Karen Blumenschein, Holly Divine

**Affiliations:** 1 St. Matthews Community Pharmacy, Louisville, KY 40207, USA; cpharlow@gmail.com; 2 American Pharmacy Services Corporation, Frankfort, KY 40601, USA; channa@apscnet.com; 3 Wheeler Pharmacy, Home Connection, Lexington, KY 40507, USA; eckmann8@aol.com; 4 General Dynamics Information Technology, Little Rock, AR 72205, USA; jane.gokun@gdit.com; 5 Department of Behavioral and Community Health, University of Maryland School of Public Health, College Park, MD 20742, USA; fzanjani@umd.edu; 6 Department of Pharmacy Practice and Science, University of Kentucky College of Pharmacy Lexington, KY 40536, USA; holly.divine@uky.edu

**Keywords:** pharmacist roles, adherence, older adults, medication management, quality of life, medication use

## Abstract

This study sought to understand the medication adherence and quality of life (QOL) of recipients of a pharmacist-based medication management program among independently living older adults. Using a cross-sectional, quasi-experimental study design, we compared older adults enrolled in the program to older adults not enrolled in the program. Data were collected via face-to-face interviews in independent-living facilities and in participants’ homes. Independently living older adults who were enrolled in the medication management program (*n* = 38) were compared to older adults not enrolled in the program (control group (*n* = 41)). All participants were asked to complete questionnaires on health-related quality of life (QOL, using the SF-36) and medication adherence (using the four-item Morisky scale). The medication management program recipients reported significantly more prescribed medications (*p* < 0.0001) and were more likely to report living alone (*p* = 0.01) than the control group. The medication management program recipients had a significantly lower SF-36 physical functioning score (*p* = 0.03) compared to the control group, although other SF-36 domains and self-reported medication adherence were similar between the groups. Despite taking more medications and more commonly living alone, independent living older adults enrolled in a pharmacist-based medication management program had similar QOL and self-reported medication adherence when compared to older adults not enrolled in the program. This study provides initial evidence for the characteristics of older adults receiving a pharmacist-based medication management program, which may contribute to prolonged independent living and positive health outcomes.

## 1. Introduction

Community pharmacists generally see patients with chronic medical conditions at least monthly, at the time medications are refilled. This regular interaction places pharmacists in a unique position to monitor and manage medications for older adults. Furthermore, extensive training in pharmacotherapy and patient communication uniquely prepares pharmacists to play a vital role in minimizing medication-related problems. Pharmacist interventions can improve patient drug knowledge and adherence [1]; however, little is known about the population that participates in pharmacist interventions, and this is especially true in the older adult population. 

A systematic review evaluating pharmacist interventions to optimize medication use in nursing home settings provided equivocal results [2]. Reviews assessing pharmacist interventions with older patients to improve health outcomes, QOL, adherence, and cost-effective care also provide mixed findings and suggest further research is needed [3,4]. Despite findings from these systematic reviews, it is clear that regular interaction and medication management provide pharmacists with the opportunity to circumvent many drug therapy problems in older adults, thereby easing patient, family, and caregiver burden. Drug therapy problems can be categorized into seven areas: unnecessary drug therapy, need of additional drug therapy, ineffective drug therapy, too low a dosage, too high a dosage, adverse drug reactions, and non-compliance [5]. Polypharmacy and drug therapy problems have been linked with poor health outcomes. For example, patients who have more medications in their home are more likely to have increased severity of their illnesses and are at higher risk for therapeutic duplications [6]. 

Humanistic parameters, such as quality of life, have been evaluated as predictors of outcomes in the older population. Health-related quality of life surveys, such as the Short Form (SF)-12 and SF-36, have been shown to be independent predictors of hospitalization and mortality [7]. These self-reported surveys provide specific feedback on patients’ physical and mental performance; a decline in these performances has been linked to a change in health status and predicts future adverse events [8]. 

Medication non-adherence has also been associated with poor health outcomes including disease progression and increased costs [9]. Research has demonstrated that a comprehensive program provided by pharmacists, including blister-packed medications, is associated with substantial improvements in medication adherence among older adults resulting in meaningful improvements in health [10]. Estimates of non-adherence in the older adult vary from 40% to 75%, and there are still many unanswered questions as to the most effective pharmacist-based intervention for promoting medication adherence [11]. 

Unfortunately, a non-invasive “gold standard” for measuring adherence is unavailable. A recent study in the United Kingdom compared three common methods for measuring adherence (electronic monitoring, pill counts, and self-report) in older adults and found substantial differences between the three methods. The use of pill counts and self-reported surveys tended to correlate better with adherence rates than prescription-bottle caps equipped with an electronic monitor that recorded when the bottles were opened. However, the inconsistency with the electronic record in the study has been postulated to be related to the varying patterns of bottle opening versus the number of pills taken out [12].

The objective of this study was to understand the medication adherence and quality of life of recipients that participate in a pharmacist-based medication management program. The ultimate goal of the service described in this study is to prolong independent living and improve overall health status for older adults. 

## 2. Materials and Methods 

### 2.1. Description of the Pharmacist-Based Medication Management Service

The pharmacist-based medication management program studied was developed specifically for independently living older adults residing either in independent living facilities or within their own home. The program is offered by a local community pharmacy. The program origins date back to the late 1980s, when a former consultant pharmacist observed that patients residing in independent and assisted living beds at a Central Kentucky nursing facility would benefit from pharmacist consultations and adherence-based services. Originally, services were only offered to residents at that one facility; however, over time, the program grew to include patients living in other facilities, and patients living in their own homes. The program was designed to allow a pharmacist to manage, monitor, and optimize medication therapy with the goal of prolonging independent living in the older patient. A key component of the program is the direct, individualized care and time devoted to each patient. Patients are referred to the program from a variety of sources ranging from self-referral to healthcare practitioner recommendation. Patients or caregivers, as well as practitioners, most often learn of the service through word-of-mouth marketing or through previous experience with the service and call the pharmacy themselves to initiate the enrollment process. Most often, these referrals are precipitated by an adverse medication event or hospitalization. 

The pharmacist-based medication management program consists of three primary components: assessment, prescription organization, and weekly support/medication dispensing. When the patient is first enrolled, initial home-based assessments are conducted to obtain baseline information for the pharmacist to better understand the patient’s specific needs. These assessments include demographic information, medication comprehension, cognitive assessments, and fall-risk assessments. This information is documented in a chart that is kept in the pharmacy, and information is updated after each weekly visit. Once the patient is enrolled into the program, all of their prescription and non-prescription medications are organized, stored at the pharmacy, and managed by a clinical pharmacist. Each week, a 7-day supply of the patient’s routine medications is prepared and delivered to the patient’s home by a pharmacy technician or pharmacist. The patient’s routine medications are dispensed in a weekly medication organizer; “as needed” medications are maintained separately. Refills are also managed through the medication management service. The pharmacist obtains prescriber authorization for refills prior to the supply of medication being depleted. If the patient has a change in a medication (for example, an increase or decrease in dose) or is started on a new medication during the week, the pharmacist on-call will be notified to dispense the medication and update the medication organizer for the patient. 

During the weekly visit, the pharmacist reviews the previous week’s medication organizer for missed doses and documents each missed dose in the patient’s chart. The pharmacist follows up with the patient or caregiver regarding patterns for missed doses and intervenes as appropriate. The pharmacist also monitors patients for other medication-related problems including adverse drug reactions, drug–drug interactions, and falls. 

Other services provided to each patient include counseling on all new or changed prescription orders, ensuring the weekly medication organizers are stored in appropriate areas of the home, an up-to-date medication list, and ongoing medication review. The clinical pharmacist is on-call 24 h per day and maintains a constant line of communication between the prescriber, the patient, and caregivers. 

### 2.2. Study Design and Population

To understand the recipients of the pharmacist-based medication management program a cross-sectional, quasi-experimental study of independently living older adults residing in central Kentucky was conducted during January and February of 2011. The study included the program recipients (intervention group), consisting of patients currently enrolled in the pharmacist-based medication management program, and a control group that was recruited for comparison. Intervention patients resided within their own home, or within independent-living facilities. The control group, living in a similar situation as the intervention group, was recruited from independently living older adults residing at three independent living facilities in the same central Kentucky town. Participants over the age of 60 and living independently were eligible for inclusion. Exclusion criteria were severe cognitive impairment (indicated by a lack of understanding of the research design and purpose of their participation) and an inability to read or write English fluently. 

The control group was recruited at three independent-living facilities in central Kentucky using flyers approved by the University of Kentucky Institutional Review Board. Control participants were asked to attend a group session for participation. The group session consisted of a description of the research, an explanation of why the participants were being asked to participate, and completion of the questionnaires. Group sessions lasted approximately 20 min. Participants were asked to complete demographic, medication adherence, and quality of life measurements. The control group completed their questionnaires and returned them to the primary investigator at the end of the live session. Intervention participants were interviewed on an individual basis in their homes. All study participants were reimbursed $15 for their time to participate. The study was approved by the University of Kentucky Institutional Review Board, and informed consent was obtained from each participant prior to participation. 

### 2.3. Measurements

A single “snap-shot” assessment of the quality of life and medication adherence was evaluated for both program participants and the control group. In this project, health-related quality of life was measured with the SF-36v2 Health Survey [13]. The SF-36v2 consists of 36 items to construct eight health domains. The domains include physical functioning (PF), role physical (RP), bodily pain (BP), general health (GH), vitality (VT), social functioning (SF), role emotional (RE), and mental health (MH). These domains are further summarized into a physical component score (PCS) and a mental component score (MCS), which have been standardized to the United States population (mean score 50; standard deviation (SD) 10). Higher scores indicate better functioning. 

**Figure 1:**
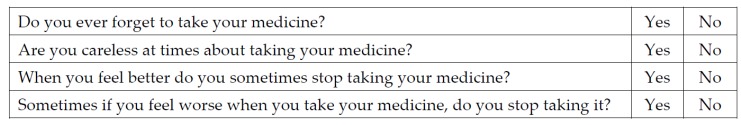
4-item Morisky scale questions [14].

Medication adherence was measured with the self-reported 4-item Morisky Scale [14], which consists of four questions assessing medication-taking behavior, as listed in Figure 1. An answer of yes to zero questions indicates high adherence behavior, answering yes to one or two questions indicates medium adherence behavior, and answering yes to three or four questions indicates low adherence behavior [14]. Medication adherence for the intervention group was also objectively measured by conducting a retrospective chart review for a total of six weeks of data. The adherence rate was calculated as a percent of medication doses actually taken divided by number of medication doses prescribed.

#### Data Analysis

Continuous variables were summarized with the use of means and SDs, or medians and interquartile range (IQR) for non-normally distributed data. The SF-36v2 data were not normally distributed, so the differences between the intervention and control groups were compared using the Wilcoxon rank-sum test. Categorical variables were summarized using descriptive statistics (counts, percentages) and compared with chi-square tests or, when appropriate, Fisher’s exact test.

## 3. Results

### 3.1. Subjects

**Figure 2:**
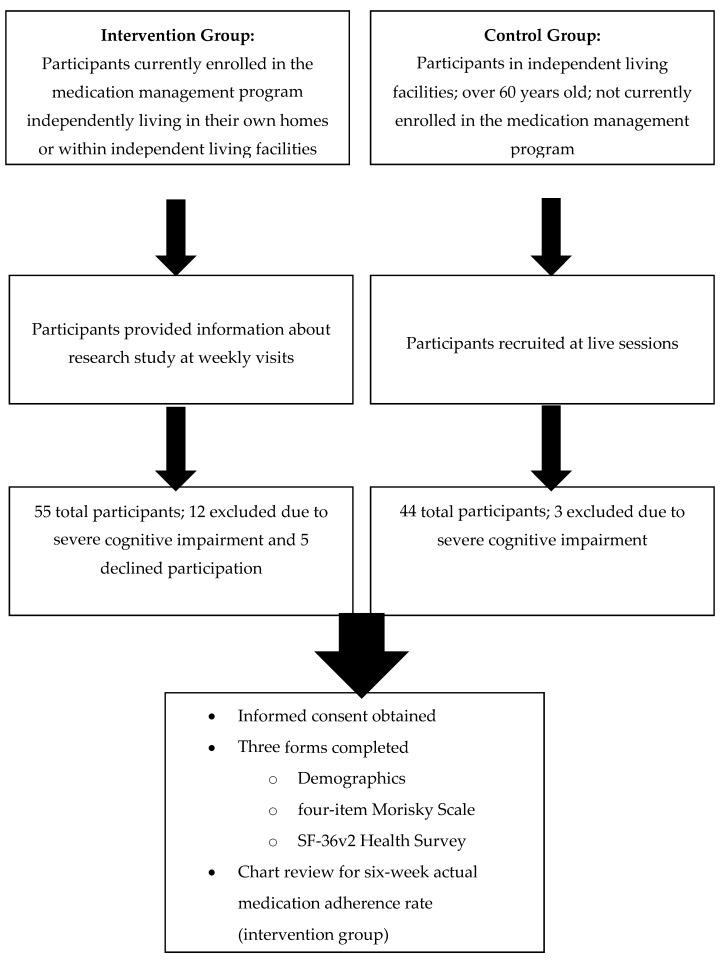
Subject Recruitment.

A total of 79 independently living older adults participated in the study. Figure 2 shows the participant recruitment flowchart. The intervention group included 38 participants and the control group included 41 participants. At the time of the study, 55 individuals were enrolled in the medication management program; 12 were excluded from the study due to severe cognitive impairment, and 5 declined participation. Three group sessions were conducted to recruit control participants. A total of 44 control participants were identified; 3 were excluded from the study due to severe cognitive impairment. 

Characteristics of the intervention and control groups are presented in Table 1. The median age for the intervention group was slightly higher compared to the control group (87 vs. 84, respectively), but this difference was not statistically significant (*p* = 0.07). Overall, the characteristics of the two study groups were similar except for the number of routine medications, number of persons living in household, and annual income. The intervention group was using significantly more regularly scheduled medications (10 vs. 5 in the control group, *p* < 0.0001), was more likely to live alone (78.9% vs. 48.8% in the control group, *p* = 0.01), and had a significantly higher annual household income (*p* = 0.001).

### 3.2. Medication Adherence

**Figure 3:**
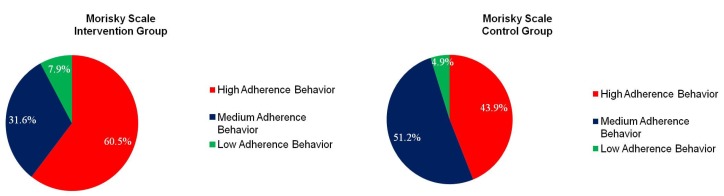
Adherence.

In the intervention group**,** 60.5% reported a high adherence rate, while 43.9% of the control group reported a high adherence rate using the self-reported four-item Morisky Scale (Figure 3), although the difference was not statistically significant (*p* = 0.21). The intervention group had a median objective medical chart adherence rate of 99% (range of 88% to 100%) during the six-week retrospective chart review (Table 2).

### 3.3. Quality of Life

With the exception of PF, there was no difference between the two groups in the eight domains of the SF-36 (Table 3). The intervention group had a significantly lower median PF compared to the control group (*p* = 0.03). 

## 4. Discussion

This evaluation of medication adherence and quality of life for independently living older adults participating in a pharmacist-based medication management program found that participants had similar QOL and self-reported medication adherence when compared to independent-living older adults not enrolled in the program, despite participants taking more prescribed medications and more commonly living alone. As previously described, medication adherence and quality of life have been shown to predict health outcomes in the older adult [1,6,7,8,9,10,11]. Adverse health outcomes result in a functional decline and a loss of independence in older adults. Interventions to improve health outcomes may reduce the risk of this loss [15,16]. Study findings demonstrate that various levels of independent living exist in the older population. It is concluded that assumptions about patient ability to manage their medications based on their level of relative “independence” should be made cautiously in the health care community.

In this study of independently living older adults, findings indicated that medication management program recipients reported being prescribed significantly more medications and were more likely to live alone. From the literature, it is well understood that taking more medications can add to the frailty and poor health outcomes of older adults. Moreover, living alone has been shown to be an independent predictor of frailty and decline in activities of daily living in older patients [17]. 

The physical functioning was significantly lower in the pharmacy program recipients, indicating a lower functionality level of independent living. All other QOL indicators were similar between the two groups. Thus, medication management program recipients have relatively similar QOL compared to other independently living older adults, but are prescribed more medications and have lower levels of physical functioning. 

Literature documents the strong positive correlation between high rates of medication adherence and positive health outcomes [9,10,18,19,20]. Although this study did not find a difference in self-reported medication adherence, the pharmacy program participants had a documented chart medication adherence rate exceeding 90%. 

As stated previously, patients are referred to medication management programs from a variety of sources, often as a result of an adverse medication event or hospitalization. Early implementation of an effective pharmacist-based medication management program could help address adverse medication-related issues and potentially prolong independent living. To improve care for the independently living older adult, it is essential to recognize the negative outcomes of suboptimal medication management. Although not directly assessed in this study, prolonged independence for the older adult reduces overall healthcare costs by preventing or delaying institutionalization. The findings of this study suggest that further evaluation of pharmacist-based medication management programs for older adults is warranted to assess potential causal relationships as well as patient, family, and caregiver perceived value of such programs. Healthcare providers should be more proactive in identifying older adults who require assistance with managing their medications and refer to pharmacists who specialize in comprehensive medication management to maintain independent living among this population.

One important limitation of this study was the difference in the recruitment process between the two groups. Participants in the intervention group were already enrolled in the medication management program possibly because they were at a higher risk for losing their independence. The research session was conducted in their home during a regular medication management program visit due to feasibility of administering the surveys. Participants in the control group attended a live session outside their home producing a different environment for completing the research questionnaires. Another limitation of the study was the cross-sectional design, which evaluated a snapshot of self-reported adherence and quality of life. Lack of baseline measures for program participants precludes an assessment of impact. Future research should study participants over time to evaluate the true impact of the medication management program on medication adherence and quality of life using a longitudinal, clinical trial design. 

## 5. Conclusions

This study provides initial evidence for characterizing older adults receiving a pharmacist-based medication management program. Individuals enrolled in the medication management program had comparable quality of life and self-reported adherence to control participants, even though the program recipients were taking more medications and were more likely to live alone. 

## Figures and Tables

**Table 1:** Characteristics of the intervention and control groups.

	Intervention Group (*n* = 38)	Control Group (*n* = 41)	*p* Value
Median Age (IQR)	87 (83–89)	84 (77–88)	0.07
Sex, Females (%)	29 (76.3%)	27 (65.9%)	0.45
**Highest level of education**			
Some college or less	21 (55.3%)	15 (36.6%)	0.22
Bachelor’s degree or equivalent	9 (23.7%)	16 (39.0%)	
Masters degree or higher	8 (21.0%)	10 (24.4%)	
Median number of regularly scheduled medications (including prescription, over-the-counter, and herbal products) (IQR)	10 (8–13)	5 (4–8)	<0.0001
Median number of disease states (IQR)	3.5 (2–5)	3 (2–5)	0.17
Trouble reading due to vision	14 (36.8%)	11 (26.8%)	0.34
Number of subjects with at least one hospital or emergency department visit in past 6 months	16 (42.1%)	15 (36.6%)	0.62
Median number of visits to hospital or emergency department in past 6 months (if greater than zero) (IQR)	1.5 (1–2.5)	1 (1–2)	0.20
Number of subjects with at least one fall in past 6 months	13 (34.2%)	14 (34.1%)	>0.90
Median number of falls in past 6 months (if greater than zero) (IQR)	1 (1–3)	1 (1–2)	0.50
**Duration of time in current home**			
0–12 months	11 (28.9%)	8 (19.5%)	0.33
1–3 years	10 (26.3%)	17 (41.5%)	
4 or more years	17 (44.7%)	16 (39%)	
**Number of people living in current household**			
1 person	30 (78.9%)	20 (48.8%)	0.01
2 persons	8 (21.1%)	21 (51.2%)	
**Annual household income**			
0–$25,000	8 (21.1%)	12 (29.2%)	
$25,001–$50,000	6 (15.7%)	17 (41.5%)	0.0014
$50,001–$100,000	3 (7.9%)	8 (19.5%)	
Greater than $100,000	13 (34.2%)	2 (4.9%)	
Not specified	8 (21.1%)	2 (4.9%)	

**Table 2:** Six-week objective medication adherence rate (%) for the intervention group (*n* = 33) *.

Mean (SD)	98 (0.03)
Median (Range)	99 (88–100)

* Five subjects were excluded from the retrospective chart review due to lack of six-week adherence information.

**Table 3:** SF-36v2 norm-based scores.

	Intervention (*n* = 38)	Control (*n* = 41)	*p* Value
Physical Component Summary (PCS)	41.77 (11.31)	45.37 (8.80)	0.12
Mental Component Summary (MCS)	55.85 (48.34–59.76)	54.67 (50.12–60.17)	0.97
Physical Functioning (PF)	36.49 (26.92–47.97)	46.06 (38.40–49.89)	0.03
Role-Physical (RP)	39.19 (32.46–54.91)	45.93 (39.19–52.66)	0.06
Bodily Pain (BP)	51.51 (42.64–62.00)	50.71 (42.64–55.55)	0.69
General Health (GH)	53.19 (43.68–57.94)	53.19 (46.05–55.56)	0.97
Vitality (VT)	52.60 (40.72–55.57)	52.60 (49.63–58.54)	0.15
Social Functioning (SF)	54.84 (37.27–57.34)	52.33 (47.31–57.34)	0.64
Role Emotional (RE)	54.43 (38.76–56.17)	52.69 (42.24–56.17)	0.97
Mental Health (MH)	53.48 (48.25–58.72)	56.10 (53.48–58.72)	0.36

Data are expressed as median (with IQR) except PCS, which is reported with mean and standard deviation; *p*-values were based on non-parametric analyses except PCS, for which a two-sample *t*-test was appropriate.
